# The relationship between mean platelet volume and thrombosis recurrence in patients diagnosed with antiphospholipid syndrome

**DOI:** 10.1007/s00296-014-2996-0

**Published:** 2014-03-27

**Authors:** Joanna Rupa-Matysek, Lidia Gil, Ewelina Wojtasińska, Katarzyna Ciepłuch, Maria Lewandowska, Mieczysław Komarnicki

**Affiliations:** Department of Haematology, Poznan University of Medical Sciences, Szamarzewskiego 84, 60-569 Poznan, Poland

**Keywords:** Antiphospholipid antibody syndrome, Mean platelet volume, Thrombosis recurrence

## Abstract

Increased mean platelet volume (MPV) is associated with platelet reactivity and is a predictor of cardiovascular risk and unprovoked venous thromboembolism. The aim of our study was to evaluate MPV in patients with confirmed antiphospholipid antibody syndrome (APS) and to identify the correlation between the value of MPV and the recurrence of thrombosis. The studied group consists of 247 patients with a history of thrombosis and/or pregnancy loss (median age 38, range 18–66 years) classified as APS group (*n* = 70) or APS negative patients (*n* = 177) according to the updated Sapporo criteria. The control group consisted of 98 healthy subjects. MPV was significantly higher in the group of patients with clinically and laboratory confirmed APS (median 7.85, range 4.73–12.2 fl) in comparison with the controls. It was also higher than in APS negative patients (7.61, range 5.21–12.3 fl). APS patients with triple positivity for antiphospholipid antibodies with respect to Miyakis classification categories had higher MPV values than other APS patients (9.69 ± 1.85 vs. 7.29 ± 1.3 fl, *p* = 0.001). Recurrent thrombotic episodes were observed in 83 patients, but among the triple positive high-risk patients with APS in 80 % cases (*p* = 0.0046). In receiver operating characteristic curve analysis, the value of MPV level for thrombosis recurrence prediction in the APS group with sensitivity of 86 % and specificity of 82 % was 7.4 fl. In the multivariate logistic regression model, MPV above 7.4 fl (OR 3.65; 95 % CI 1.38–9.64, *p* = 0.009) significantly predicts thrombosis recurrence. Our results identify the value of MPV as a prognostic factor of thrombosis recurrence in patients with APS.

## Introduction

Antiphospholipid antibody syndrome (APS) is an acquired thrombophilic condition associated with venous and/or arterial thrombosis and/or pregnancy morbidity with the presence and persistence of antiphospholipid antibodies (Abs). The average population prevalence of Abs in asymptomatic subjects ranges from 1 to 5.6 % [1, 2]. The laboratory diagnosis of APS is based on tests identifying the presence of Abs such as anti-β2 glycoprotein-I (anti-β2GPI) antibodies, anticardiolipin (aCL) antibodies or lupus anticoagulant (LA) on two or more occasions at least 12 weeks apart [3].

Although the mechanism of thrombosis in patients with Abs is not completely known, several mechanisms have been proposed including Abs interference with endogenous anticoagulant mechanisms (i.e. disruption of the annexin A5 activity, inhibition of the protein C pathway), interacting with endothelial cells and inducing increased expression of adhesion molecules and tissue factor or activation of the complement cascade. Increasing platelet activation, adhesion and aggregation play important roles in a prothrombotic state [4–6]. Additionally, recurrent thrombosis is observed only in some patients with APS, which may suggest different mechanisms of hypercoagulable processes in different patients.

One of the currently discussed mechanisms related to a high risk of thromboembolism is increased mean platelet volume (MPV), as has been reported in meta-analysis and prospective studies [7, 8].

Therefore, the question arises whether MPV is directly or indirectly connected with thrombosis occurrence in APS patients. To our knowledge, little is known about the value of MPV and its prognostic significance for thrombosis recurrence in patients with confirmed antiphospholipid syndrome.

The aim of this study was to evaluate and compare MPV in patients diagnosed with APS to patients with thrombotic complications without APS or healthy controls and to identify the correlation between the value of MPV and thrombosis recurrence.

## Methods

A total number of 247 patients with a history of thrombotic episodes and/or pregnancy loss were analysed between 2009 and 2012. All patients were white Caucasians with median age 38 (range 18–66) years. For all patients, demographical data, clinical risk factors for arterial and venous thrombosis and smoking status were analysed (Table 1). Patients with malignancy were excluded from the analyses.

**Table 1:** Demographical and clinical characteristics of the studied patients

Characteristics	*n* = 247
Age, years, median (range)	38 (18–66)
Sex, female/male (*n*)	160/87
Risk factors for venous thrombosis (*n*)	
Surgery, immobilization or trauma	12
Immobilization or trauma	12
Use of oral contraceptives or hormone replacement therapy	73
Pregnancy or postpartum period until 6 weeks	16
Family history	20
Risk factors for arterial thrombosis (*n*)	
Diabetes mellitus	47
Hypertension	74
Hypercholesterolaemia	90
Obesity	89
Smoking habit	64
Family history	35

APS and other hereditary thrombophilia tests were performed on all studied patients. For coagulation tests, patients were recruited at least 6 months after a thrombotic event and at least 4 weeks after anticoagulation with vitamin K antagonists had been discontinued.

For APS, LA, measurement of IgG and IgM aCL antibodies and anti-β2 glycoprotein-I (aβ2GPI) were performed according to the updated Sapporo classification criteria with respect to the manufacturer’s recommendations (Siemens Healthcare Diagnostics Products GmbH, Marburg, Germany). Cardiolipin and anti-β2 glycoprotein-I antibodies assays were calibrated against Sapporo Standard (INOVA Diagnostics, San Diego, USA).

Additional thrombophilia tests included deficiencies of antithrombin, protein C and protein S, activated protein C resistance assays (APCr-PCA ratio) and increased plasma level of factor VIII (Helena Biosciences Europe, UK, Gateshead). Factor V Leiden G1691A and prothrombin G20210A polymorphisms were determined as described elsewhere [9, 10]. D-dimer and homocysteine were analysed by routine laboratory techniques using Siemens Healthcare Diagnostics Products.

To find the influence of MPV on thrombosis risk and thrombosis recurrence, this parameter was analysed in the APS patients and in the APS negative group, as well as in healthy subjects. The control group consisted of 98 age-matched healthy subjects, median age 41 (range 20–63 years); 60 % were male. None of them had personal history of VTE or arterial thrombosis (myocardial infarction or stroke).

For MPV estimation, a full blood count was performed on an automated analyser within 60 min of collection by a CELL-DYN 3700SL analyser using impedance technology (Abbott Diagnostics, Chicago, USA) [11]. Fasting blood samples were collected into vacuum tubes with minimal stasis (Sarstedt, Nümbrecht, Germany).

The Local Bioethical Committee approved this research protocol.

### Statistical methods

The results are presented using methods of descriptive statistics such as frequency (*n*), arithmetic mean ($\overline{x}$) and standard deviation (SD). The Shapiro–Wilk’s test was performed to assess normality. In order to compare differences between the groups, the Chi-square test or Fisher’s exact test was used for categorical variables and the Mann–Whitney *U* test for continuous variables. For variables with a normal distribution and homogeneous variances, the significance of differences between the groups was examined by analysis of variance followed by the Tukey’s post hoc test. For variables not normally distributed or of unequal variance, ANOVA analysis of variances followed by the nonparametric Kruskal–Wallis’s test was applied.

Correlations between the individual parameters and MPV were calculated using the Spearman rank correlation method. Multiple regression was performed to investigate which Abs caused high MPV in APS patients with triple positivity. The coefficient of determination was used to evaluate how well the model fits the obtained data (R-Square).

Receiver operating characteristic (ROC) analysis was performed to determine the sensitivity and specificity with 95 % confidence intervals (CIs) at cut-off values for the MPV level predictive of thrombosis recurrence.

A multivariate logistic regression was used to evaluate potential risk factors that might influence thrombosis recurrence. In each model, the odds ratio (OR) for each independent variable with a 95 % confidence interval (CI) was determined. A *p* value below 0.05 was regarded as statistically significant. The statistical analyses were performed with STATISTICA 10 and STATISTICA Medical Package 2.0 (StatSoft, Inc. 2013 software).

## Results

Antiphospholipid antibody syndrome was diagnosed in 70 patients (median age 37 years, and 37 % of whom were male) in the absence of any underlying autoimmune disease. All of them fulfilled both clinical and laboratory criteria for APS diagnosis according to Miyakis [3] (APS patients). One hundred and seventy-seven patients with a history of thrombotic events, either venous thromboembolism or arterial thrombosis, in whom the presence of Abs was not confirmed, were placed in the APS negative group (APS negative patients).

Of the 57 DVT episodes in the APS patients, 21 (39.62 %) had proximal DVT alone, 14 (26.41 %) had symptomatic pulmonary embolism (PE), nine cases had DVT combined with PE and 20 cases were diagnosed with distal DVT. Among the patients with cerebral thrombotic episodes (*n* = 11), the most common were ischemic stroke/transient ischemic attack (82 %). Pregnancy morbidity according to classification criteria for APS was recorded in ten women in the APS group [3]. Among women, a history of oral contraceptives was identified in 73 of all cases (45.6 %), ten in the APS group. Pregnancy/postpartum thrombotic complications were identified in 4 (9 %) of the APS women.

The main characteristics of patients and frequency of clinical thrombotic manifestations associated with APS are shown in the Table 2.

**Table 2:** Clinical characteristics of the APS positive and negative patients

Characteristics	*n*	APS patients (*n* = 70)	APS negative patients (*n* = 177)	*p* value
Female/male gender (*n*)	160/87	44/26	116/61	0.852
Median age, years (range)	247	37.0 (18–66)	38.1 (18–65)	0.478
Type of thrombosis [no. (%)]				
Proximal deep vein thrombosis	105	21 (30.0)	84 (47.45)	**0.007**
Pulmonary embolism	44	14 (20.0)	30 (16.95)	0.762
Cerebral vein thrombosis/stroke	55	11 (15.4)	44 (24.86)	0.192
Pregnancy loss	26	10/44 (22.72)	16/116 (13.79)	0.151
Distal deep vein thrombosis	51	20 (28.57)	33 (18.64)	0.341
Inherited thrombophilia [no. (%)]				
Antithrombin deficiency	6	4 (5.71)	2 (1.1)	**0.002**
Protein C deficiency	5	2 (2.86)	3 (1.7)	0.219
Protein S deficiency	2	1 (1.43)	1 (0.56)	0.256
Factor V G1691A	37	12 (17.14)	25 (14.12)	0.086
Prothrombin G20210A	8	1 (1.43)	7 (3.95)	0.516

According to the Miyakis laboratory criteria, the APS patients were allocated to category I or category II on the basis of positivity to Abs [3]. Single test positivity was found in 48 cases, which constitute 68 % of APS patients, category IIA (only LA detection). Triple Abs positivity (category I) defined by the presence of LA and high titres of IgG or IgM aCL > 40 GPL/MPL and IgG or IgM anti-β2GPI > 99th percentile antibodies was identified in ten patients with APS (14.28 %), while double positivity (also category I) in 12 cases of confirmed APS—17.14 %.

All three groups, the APS, the APS negative and the controls did not differ with respect to age and platelet counts. MPV was significantly higher in the APS group (mean 7.85, range 4.73–12.2 fl) in comparison with the controls (6.85, range 5.01–7.93 fl, *p* = 0.004). There was no difference in regard to MPV between the APS group (mean 7.85, range 4.73–12.2 fl) and the controls (6.85, range 5.01–7.93 fl, *p* = 0.004), Table 3.

**Table 3:** Characteristics of patients with or without APS, and the healthy controls

Characteristics	APS positive patients (*n* = 70)	APS negative patients (*n* = 177)	Controls (*n* = 98)	*p* value
Age (years)	38.6 ± 13.8	40.2 ± 12.7	40.3 ± 12.6	0.677
Female/male (*n*)	44/26	116/61	37/61	**0.001**
Platelet parameters				
PLT (×10^9^/L)	257.0 ± 68.4	227.2 ± 72.1	263.4 ± 55.9	0.219
MPV (fl)	7.85 ± 1.81	7.61 ± 1.21	6.85 ± 0.71	<0.001*

Values are expressed as mean $\overline{x}$ ± SD or percentage

*PLT* platelet count, *MPV* mean platelet volume

* Post hoc (APS positive patients vs. controls *p* = 0.002 and APS negative group vs. controls *p* ≤ 0.001)

A significant positive correlation was found between the value of MPV and Abs titre, with the Spearman correlation coefficients of 0.018 (*p* = 0.0116) for the increased ratio of LA1/LA2 and 0.018 (*p* = 0.0082) for anti-β2 glycoprotein-I. No significant correlation was evident between MPV and aCL antibodies.

**Fig. 1:**
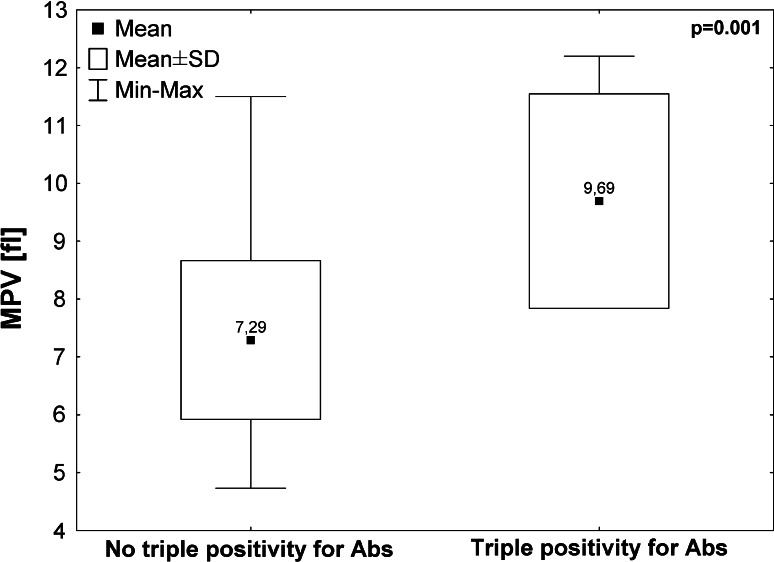
MPV levels in patients with triple Abs positivity and other Miyakis classification categories for APS

With respect to MPV, a significantly higher MPV was found in patients with triple positivity for Abs in comparison with other Miyakis classification categories (9.69 ± 1.85 vs. 7.29 ± 1.3 fl, *p* = 0.001), Fig. 1. The median platelet count was 230 (range 126–301 G/L) for the patients with triple positivity for Abs and 267 (range 123–418 G/L) for all the other APS patients. In multiple regression, the analysed parameters, including LA, aCL and aβ2GPI (IgG and IgM), had an effect on the value of MPV in patients with triple positivity for Abs. The model explained 85 % of the variability of MPV (*F* = 7.46, *p* < 0.0244). In this model, the ratio of LA1/LA2 and the level of aβ2GPI were proven to be statistically significant (*p* = 0.0203 and *p* = 0.0081).

### Cut-off value of MPV for prediction of thrombosis recurrence

**Fig. 2:**
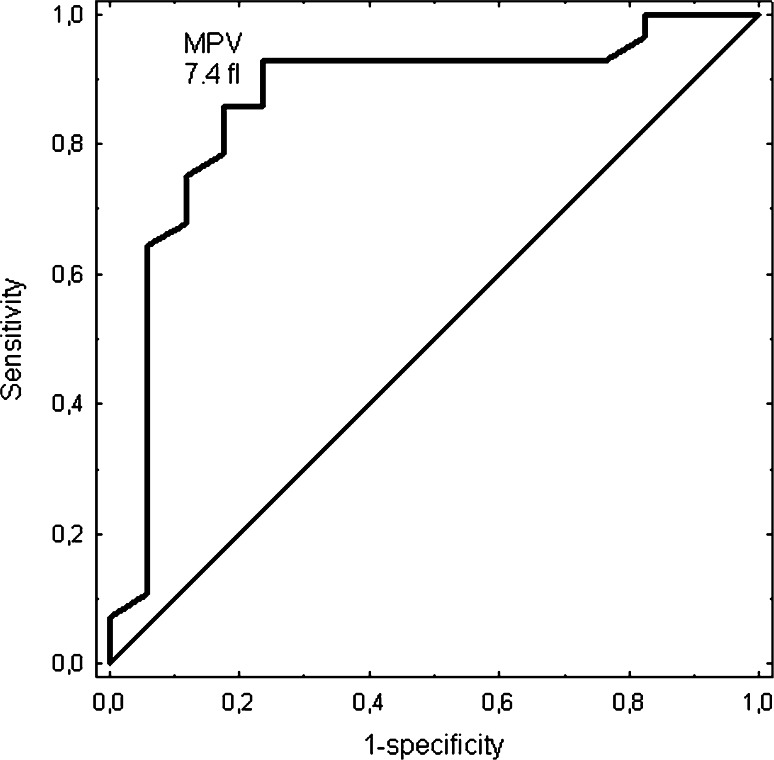
ROC curve of MPV for thrombosis recurrence prediction in APS group. Area under the curve (*AUC*) 0.863. CI (95 %) 0.739–0.988. SE 0.064

The ROC analysis indicated a cut-off value of 7.4 fl for MPV with 86 % sensitivity and 82 % specificity (AUC = 0.863; *p* = 0.0001) for thrombosis recurrence prediction (Fig. 2).

### Factors affecting thrombosis recurrence

Recurrent thrombosis was observed in 83 patients of the studied cohort (*n* = 247). Among the APS positive patients, recurrent thrombotic episodes were recorded in 66.26 % (55/70). In 18 cases, multiple thrombotic events were present. Venous thromboembolism recurrence was recorded in 42 cases (60 %), recurrence of arterial thrombosis in 11 patients (15.71 %) and both in two patients (2.86 %) with confirmed APS, while in the APS patients with triple positivity for Abs, recurrent thrombotic events were recorded in ten cases (80 %, *p* = 0.0046). One patient died in the course of thrombosis recurrence complication, this patient was also a homozygous carrier of factor V Leiden G1691A mutation and had a history of both venous and arterial thrombotic episodes.

The risk-factor analysis for thrombosis recurrence in the APS group included age, male gender, history of PE, hereditary thrombophilia and the following laboratory parameters; MPV (≥7.4 fl), factor VIII activity and D-dimer level. Upon univariate analysis, MPV (≥7.4 fl), previous PE history and hereditary thrombophilia were the only factors significantly associated with the risk of thrombosis recurrence in our cohort. In the multivariate logistic regression model, only MPV above 7.4 fl (OR 3.65; 95 % CI 1.38–9.64, *p* = 0.009) significantly affected the risk of thrombosis recurrence in the APS group (Table 4).

**Table 4:** Univariate and multivariate analyses determining predictors for thrombosis recurrence in patients with APS

Factor	Univariate analysis OR (95 % CI), *p*	Multivariate analysis OR (95 % CI), *p*
Male gender	1.83 (0.33–10.22), *p* = 0.338	0.65 (0.24–1.77), *p* = 0.394
Age	0.53 (0.06–4.84), * p* = 0.562	0.99 (0.91–1.01), *p* = 0.823
Previous pulmonary embolism history	5.62 (1.07–29.42), *p* = 0.041	0.36 (0.09–1.44), *p* = 0.364
Hereditary thrombophilia	5.63 (1.08–29.41), * p* = 0.040	3.328 (0.57–19.46), *p* = 0.182
Elevated factor VIII	1.02 (0.99–1.03), * p* = 0.322	0.98 (0.94–1.01), *p* = 0.224
Elevated D-Dimer	1.01 (0.99–1.01), * p* = 0.873	1.0 (0.998–1.00), *p* = 0.513
MPV ≥ 7.4 fl	42.25 (6.82–261.63), *p* = 0.001	3.65 (1.38–9.64), *p* = 0.009

*MPV* mean platelet volume

## Discussion

Among many mechanisms of thrombosis in patients with antiphospholipid syndrome, the pivotal role of platelets is well established. It is known that platelet activation and aggregation predispose to thrombotic events in patients with antiphospholipid syndrome [6]. There has been evidence that Abs enhance platelet activation through the apolipoprotein ER2 receptor and therefore activate the p38 mitogen-activated protein kinase (p38MAPK) pathway with phosphorylation of cytosolic phospholipase A2 (cPLA2) ending in thromboxane B2 production, and this results in platelet aggregation, especially by increasing the expression of GPIIb–IIIa [12].

The platelet size, measured as MPV, reflects platelet reactivity, including aggregation, glycoprotein IIb-IIIa expression and production of more thrombogenic factors (thromboxane A2, platelet factor 4, β-thromboglobulin, P-selectin and platelet-derived growth factor) [11, 13, 14]. The regulation of the platelet size is multifactorial and independent of the age of platelets [8]. Evidence from meta-analysis and prospective studies indicated that an increased MPV is not only a predictor of unprovoked venous thromboembolism [7], but also a predictor of cardiovascular risk including acute myocardial infarction, its mortality and restenosis following coronary angioplasty [8, 15]. Besides this, there are reports concerning the association between increased MPV and heart failure, arterial stiffness, acute PE, stroke prediction and mortality [16–19].

So far, little is known about the mean value of MPV in patients diagnosed with APS. The available data are inconsistent. In one report comparing two different haematological analysers, lower MPV was recorded in 14.28 % and in 65.71 % of cases with APS (*n* = 35), but a relation with thrombotic potency was not studied [20]. A more recent study, however, reported that among patients with thrombocytopenia, five in nine patients with antiphospholipid syndrome had increased MPV [21].

To establish if MPV might be an indirect indicator of platelet activation in the APS, we analysed MPV in patients with confirmed antiphospholipid syndrome and in patients with thrombotic complication without APS, as well as in asymptomatic controls. In our cohort of patients, MPV was statistically significantly higher in the APS patients in comparison with the control subjects. It also tended to be higher in the APS group when compared with the APS negative group. A significantly positive correlation between LA and aβ2GPI for the value of MPV was observed in the APS group, but the reason for this fact is unknown.

Triple positivity for Abs, which is described by Vitorio Pengo’s group as being associated with high thrombotic recurrence rates in Italian APS patients, is also associated with high thrombotic recurrence rates in our cohort [22, 23]. APS patients with triple positivity for Abs have significantly higher MPV values than other APS patients. In multiple regression, the presence of LA and aβ2GPI contributed to the prediction of the high MPV values in APS group with triple positivity for Abs.

We have also analysed the impact of the value of MPV on thrombosis recurrence in the analysed patients cohort with APS. A number of risk factors for arterial and/or venous thrombosis recurrence have already been defined [1, 24, 25]. Increased MPV has been noted in subjects with cardiovascular risk factors, such as smoking, diabetes, obesity, hypertension and hyperlipidaemia [26]. The risk of thrombosis recurrence in our study in a univariate analysis was significantly related to the value of MPV. Moreover, in the case of multivariate analysis, it was found that the probability of thrombosis recurrence was higher in the studied patients with APS. A level of MPV above 7.4 fl was identified as a prognostic factor of thrombosis recurrence in patients with APS.

Our results are in line with the study by Korkmaz et al. [27] on 22 patients with antiphospholipid syndrome and 22 healthy controls that showed higher MPV in both patients with primary and secondary APS.

We found the coexistence of a moderate antithrombin deficiency with antiphospholipid syndrome in four cases. It may be a laboratory inaccuracy due to antithrombin inhibition by Abs [28].

Our study has several limitations. The number of study participants was limited. Besides, this was a one-centre study using one type of automated analyser. The use of different automated cell counters might influence measurement and final results [29].

In conclusion, our study identifies the value of MPV as a prognostic factor of thrombosis recurrence in patients with APS. The results support the assumption that increased MPV could be an indirect indicator of platelet activation in patients with APS. In all likelihood, it may identify a group at the highest risk of thrombotic complications, as confirmed in our statistical analysis. The question of whether MPV may be a potentially useful prognostic biomarker for the prediction of recurrent venous or arterial thrombotic events in patients with antiphospholipid syndrome should be clarified in further prospective studies.
